# Psycho-Socio-Economic Issues Challenging Multidrug Resistant Tuberculosis Patients: A Systematic Review

**DOI:** 10.1371/journal.pone.0147397

**Published:** 2016-01-25

**Authors:** Beena Elizabeth Thomas, Poonguzhali Shanmugam, Muniyandi Malaisamy, Senthanro Ovung, Chandra Suresh, Ramnath Subbaraman, Srividya Adinarayanan, Karikalan Nagarajan

**Affiliations:** 1 National Institute for Research in Tuberculosis (formerly Tuberculosis Research Centre), ICMR, Chennai, India; 2 Brigham and Women’s Hospital, Harvard Medical School, Boston, MA, United States of America; 3 Vector Control Research Centre, ICMR, Pondicherry, India; Public Health Research Institute at RBHS, UNITED STATES

## Abstract

**Background:**

Limited treatment options, long duration of treatment and associated toxicity adversely impact the physical and mental well-being of multidrug-resistant tuberculosis (MDR-TB) patients. Despite research advances in the microbiological and clinical aspects of MDR-TB, research on the psychosocial context of MDR-TB is limited and less understood.

**Methodology:**

We searched the databases of PubMed, MEDLINE, Embase and Google Scholar to retrieve all published articles. The final manuscripts included in the review were those with a primary focus on psychosocial issues of MDR-TB patients. These were assessed and the information was thematically extracted on the study objective, methodology used, key findings, and their implications. Intervention studies were evaluated using components of the methodological and quality rating scale. Due to the limited number of studies and the multiple methodologies employed in the observational studies, we summarized these studies using a narrative approach, rather than conducting a formal meta-analysis. We used ‘thematic synthesis’ method for extracting qualitative evidences and systematically organised to broader descriptive themes.

**Results:**

A total of 282 published articles were retrieved, of which 15 articles were chosen for full text review based on the inclusion criteria. Six were qualitative studies; one was a mixed methods study; and eight were quantitative studies. The included studies were divided into the following issues affecting MDR-TB patients: a) psychological issues b) social issues and economic issues c) psychosocial interventions. It was found that all studies have documented range of psychosocial and economic challenges experienced by MDR-TB patients. Depression, stigma, discrimination, side effects of the drugs causing psychological distress, and the financial constraints due to MDR-TB were some of the common issues reported in the studies. There were few intervention studies which addressed these psychosocial issues most of which were small pilot studies. There is dearth of large scale randomized psychosocial intervention studies that can be scaled up to strengthen management of MDR-TB patients which is crucial for the TB control programme.

**Conclusion:**

This review has captured the psychosocial and economic issues challenging MDR patients. However there is urgent need for feasible, innovative psychosocial and economic intervention studies that help to equip MDR-TB patients cope with their illness, improve treatment adherence, treatment outcomes and the overall quality of life of MDR-TB patients.

## Introduction

The emergence of strains with MDR-TB has led to a resurgence of TB as a major public health menace worldwide. It has been reported that about 3.6% of new TB patients in the world have MDR-TB and the levels have been found to be as high as 20% in previously treated TB patients. China, India and Russia are estimated to bear about 60% of the global burden of MDR-TB disease [1]. Most MDR-TB patients remain undetected and untreated [2,3], exposing their families and communities to the risk of acquiring MDR-TB strains transmitted through the air especially in high density communities and among people with HIV/AIDS [1,4].

This emergence of MDR-TB is attributed primarily to poor patient management and non-adherence to the prescribed regimen [5,6]. Globally, the proportion of MDR-TB patients who successfully complete treatment remains less than 50%. These poor outcomes are primarily due to the long duration of treatment (often 24 months or longer) and drug toxicity [7], which result in poor treatment adherence. Furthermore psychosocial issues often complicate MDR-TB, given the complexity and long duration of treatment [8, 9]. Indiscriminate prescription practices [10], by private practitioners and inability of patients to afford the complete course of treatment offered by the private clinicians has added to the problem of MDR-TB [11], Despite research advances in the microbiological and clinical aspects of MDR-TB, research on the psychosocial context of MDR-TB is limited and less understood. In this paper, we review the psychosocial challenges faced by MDR-TB patients which complicates the MDR-TB treatment and have also reported psychosocial interventions that have been implemented to address these issues.

## Methods

This systematic review has been conducted according to the PRISMA guidelines. Prior to this study no protocol was developed (S1 Table).

### Search criteria

We searched PubMed, Embase, and Google Scholar to identify all published articles using the following key words: tuberculosis, resistant tuberculosis, multidrug resistant tuberculosis, psychological factors, social factors, psychosocial issues and socio-economic factors. Zotero, open source reference management software, was used to cite and manage the data by online library programme.

### Selection of studies

Out of the 282 articles retrieved using the search key words, selection was done using the following inclusion and exclusion criteria.

#### Inclusion criteria

Both quantitative and qualitative studies were included if they assessed the psychosocial issues of adult MDR-TB patients or if they reported interventions to address psychosocial issues and outcome of interventions from different countries. Psychosocial indicators related to MDR-TB specifically included depression, stigma, anxiety, disclosure issues, economic constraints, treatment default, alcohol dependence and quality of life. Studies in the English language or with an English-language translation available were included in the final list.

#### Exclusion criteria

Studies on drug-susceptible TB, clinical studies of MDR-TB (e.g., clinical aspects, treatment management, and treatment outcomes), pharmacology of MDR-TB medications, and epidemiology of MDR-TB were excluded. Studies involving children were excluded (S2 Table).

### Data extraction

After completion of the search process, selection of articles for inclusion and extraction of key findings from the studies were conducted independently by two reviewers (PS and MM). Disagreements during study selection or data extraction were resolved by a third reviewer (BT). Then the final set of manuscripts was assessed by the research team and information was thematically extracted on the objective of the study, the methodology (quantitative or qualitative) employed, the study setting, salient findings, any interventions performed and implications or recommendations resulting from the study findings.

During data extraction, we classified the study findings into the following categories: (1) psychological issues, (2) social issues which included economic issues and (3) psychosocial interventions to improve quality-of-life for MDR-TB patients. We defined “psychological issues” as including any adverse impact on the mental state of the patients resulting either from the diagnosis of MDR-TB or from MDR-TB medications; we also included the quality of life of the patients in this context. We defined “social issues” as including any problems faced by the patients in their interactions with family members or the local community due to MDR-TB; in particular, this includes problems such as stigma, discrimination, and lack of social support. We included drug and alcohol dependence in this category, as these are behavioural issues influenced by society [12,13]. Furthermore, economic and financial challenges faced by patients and their families were included in the category of social issues. “Psychosocial interventions” were considered as including any interventions that addressed any of the psychological, social, or economic problems faced by MDR-TB patients with the goal of improving either treatment outcomes or quality-of-life.

### Analysis

Due to the limited number of studies and the multiple methodologies employed in the observational studies, we summarized these studies using a narrative approach, rather than conducting a formal meta-analysis. We used ‘thematic synthesis’ method for extracting qualitative evidences and systematically organised to broader descriptive themes [14], which were further compared for interrelations and classified into three final themes. For the intervention studies with an experimental design, we rated the quality of each intervention using components of the Methodological Quality Rating Scale (MQRS) [15]. While the MQRS includes 12 quality indicators, the methodologies of the intervention studies were so diverse that only three of these indicators namely group allocation, quality control, and follow-up rate could be applied across all studies (Table 1). In addition to these three indicators of MQRS we also added one more indicator. This indicator assessed the association of the psychosocial intervention with MDR-TB treatment outcomes. We classified the intervention papers into two categories. Some studies described psychosocial benefits resulting from specific programmatic models of MDR-TB care but did not contain experimental research designs; we describe these studies in a section entitled “Descriptions of programmatic models of MDR-TB care”. Other studies described the results of psychosocial interventions that were tested using experimental designs; we described these results in a section titled “Intervention studies with experimental designs”.

**Table 1:** Quality rating system for the psychosocial intervention studies.

Scales	Scoring	Baral et al.	Kaliakbarova et al.
**Group allocation**		0 (Low)	1 (Low)
High = 4	4 = Randomization		
High = 3	3 = Within subject counterbalanced		
Medium = 2	2 = Case control / matching		
Low = 1	1 = Quasi-experimental design, arbitrary assignment, sequential cohorts		
Low = 0	0 = Violated randomization or non-equivalent groups or pilot intervention		
**Quality control**		1 (High)	1 (High)
High = 1	1 = Treatment standardized by manual, specific training, content coding, etc.		
Low = 0	0 = No standardization of treatment specified		
**Follow-up rate**		1 (Medium)	2 (High)
High = 2	2 = 85–100% follow-ups complete		
Medium = 1	1 = 70–84.9% follow-ups complete		
Low = 0	0 = <70% follow-ups complete or longest follow-up < 3 months		
**Link to treatment outcome**		1 (High)	1 (High)
High = 1	1- Linked to treatment outcome		
Medium = 0	0- Not linked to treatment outcome		

Note: This rating system was only applied to the two intervention studies with an experimental design.

## Results

### Number of studies selected

**Fig 1:**
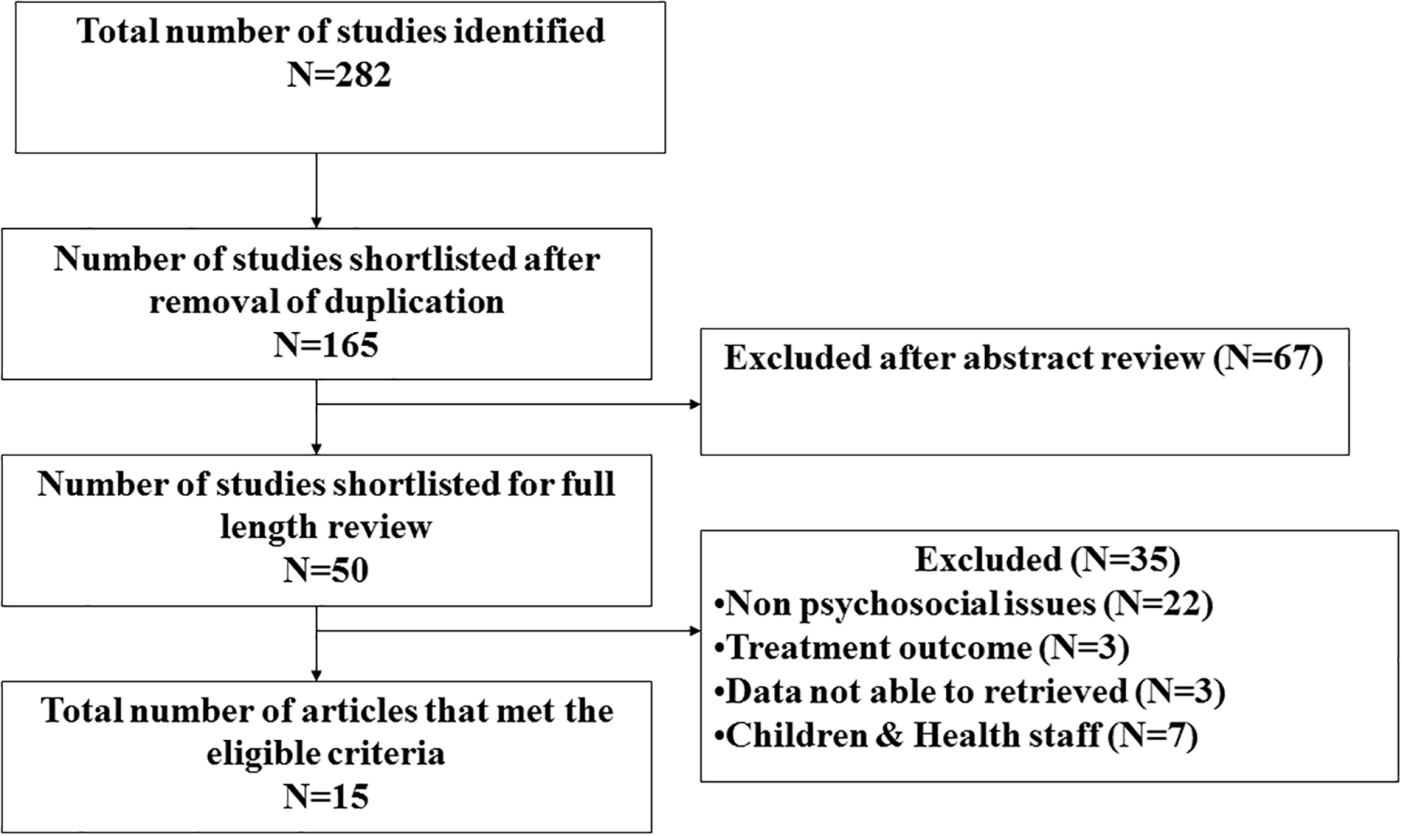
Flow diagram indicating the process of selecting the studies for this systematic review on the psychosocial issues associated with MDR-TB.

Fig 1 depicts the study selection process. The literature search from all sources yielded 282 citations. Of these, 15 articles with the primary objective of assessing the psychosocial issues of MDR-TB patients were included in the analysis.

### Study settings and characteristics

**Fig 2:**
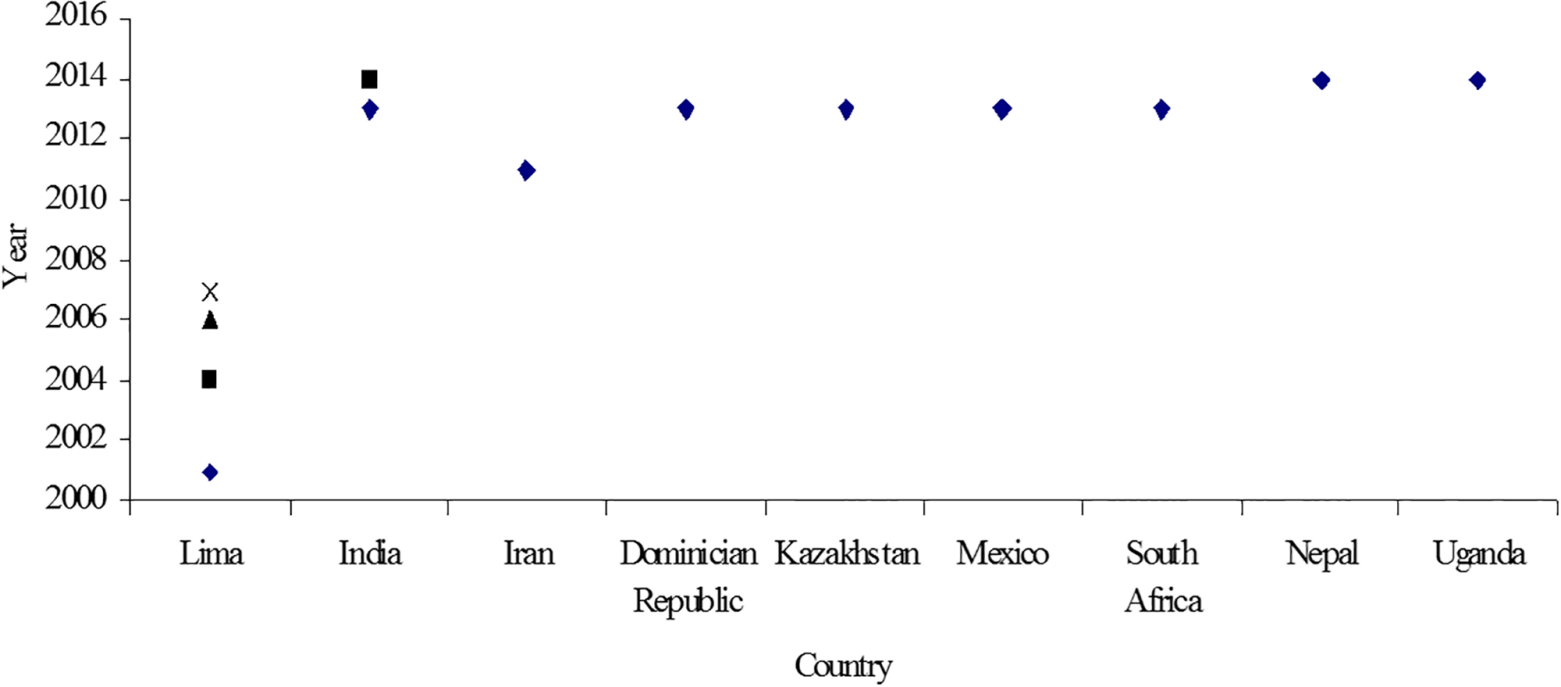
Publications on psychosocial issues of MDR-TB in different regions in different periods.

Among the 15 studies selected (Fig 2), a total of five studies were conducted in Lima, Peru between 1996–2004 [7,8,16,17,18]. Three studies have been conducted in India, of which two studies were undertaken as part of a HIV/MDR-TB intervention during 2012–2014, [19,20] and another study was conducted in the general MDR-TB population in a hospital in North India [21]. The remaining seven studies were conducted in different countries including Nepal [22] in 2008, East Kazakhstan [23] in 2010, Mexico [9] in 2009, South Africa in 2007–2010 [24], North Uganda in 2001 [25], Iran in 2006–2009 [26] and Dominician Republic in 2013 [27]. Out of the total fifteen studies, two were conducted exclusively in rural areas of sub-Saharan Africa [24,25,26,27], and ten were conducted in metropolitan cities or national/regional capitals, specifically Lima (Peru), Mumbai (India), Kathmandu (Nepal) and Tehran (Iran) [7,8,16,17,18,19,20,22,26,27]. One study alone was conducted with study participants from two different countries, USA and Mexico [9]. The remaining two studies have not clearly mentioned the study settings [21,23].

Of the 15 studies, 6 were qualitative studies [8,9,17,18,19,25], one was a mixed methods study employing both qualitative and quantitative designs [22], and 8 were conducted using a quantitative design. Of the studies primarily using a quantitative methodology, four were retrospective cohort studies [7,16,20,24], two were prospective cohort studies [23,26] and two were cross sectional studies [21]. All the 15 studies were broadly focused on the following issues faced by MDR-TB patients: (1) psychological issues, (2) social issues which also covered the economic issues and (3) psychosocial interventions to improve MDR-TB care.

### (1) Psychological issues of MDR-TB patients

Eight studies investigated the psychological issues faced by the MDR-TB patients (Table 2). They have demonstrated that the most common emotion expressed by patient is the feeling of hopelessness and fear, once diagnosed with MDR-TB [9,19]. This is reflected in the feeling that MDR-TB treatment is the final option for them, which corroborates their inherent fears of the effectiveness of MDR-TB treatment in offering them cure for their disease [8,17,19]. Studies have also reported a perceived loss of self-identity, low self-esteem, feeling of guilt, isolation and depression for the majority of the MDR-TB patients [8,9,17,18,19,21,22]. The studies also report that patients often feel distressed when changing their life style to accommodate the long course of therapy required for MDR-TB. Many patients reported living an “isolated life” and lack of participation in their normal routine which resulted in making them feel “less valuable”, sometimes leading to depression [9]. One study reported that having more dependent children was a risk factor for anxiety, and the stress of being able to fulfil a care giving role while being sick [7]. Another study assessed the quality of life of MDR-TB patients in terms of physical, psychological, environmental and social domains. This study found that mean quality of life score of MDR-TB patients was more affected when compared to drug-susceptible TB patients in the psychological domain (15 vs 17), the social domain (8 vs 10) and the environmental domain (15 vs 19) [21], with a lower score indicating lower quality of life.

**Table 2:** Observational studies on psychosocial issues of MDR-TB patients.

Reference	Year	Place	Method	Popn.	Objective	Findings	Implications
Isaakidis et al. [19]	2013	India	Qualitative	12	Factors influencing treatment adherence	Patients voiced concerns about stigma, depression, hopelessness about the efficacy of treatment, guilt due to lack of work productivity, lack of emotional support, and the perception that the side effects of medications cause greater suffering than the disease.	Family care givers were crucial in providing emotional and psychological support for the MDR-TB patients.
Morris et al. [9]	2013	Mexico	Qualitative	12	Psychological, social & economic effects	Patients reported suffering from stigma, discrimination, voluntary separation from their families, loss of self-identity and self-esteem, financial hardship, and isolation.	Psychological, social, and economic support interventions need to be included as a regular part of MDR-TB care.
Kendall et al. [24]	2013	South Africa	Retrospective	225	Risk factors for treatment default	Younger, economically unstable patients, and alcohol and drug users were particularly at risk for default. Formal housing (hazard ratio 0.38) and steady employment (hazard ratio 0.41) were associated with decreased risk of default.	Psychological and socioeconomic support are crucial for improving treatment outcomes, especially formal housing and employment.
Baghaei et al. [26]	2011	Iran	Cohort (Intervention)	80	Adverse effects	Patient had a high rate of adverse effects from MDR-TB medications, including neuropsychiatric side effects like depression, altered consciousness, convulsions, and suicide in 7.5%. Patients with neuropsychiatric side effects had a statistically significant less favorable outcome (p = 0.038).	These is an urgent need for strategies to manage adverse effects due to MDR-TB therapy.
Sharma et al. [21]	2013	India	Cross sectional	60	The impact on quality of life	The mean quality of life scores for MDR-TB patients were statistically significantly lower across all domains (psychological, social and environmental) when compared to patients with drug-susceptible TB.	MDR-TB patients have a greater need for psychosocial support when compared to patients with drug-susceptible TB.
Mauch et al. [27]	2013	Dominician Republic	Cross sectional	20	Costs incurred by MDR-TB patients	MDR-TB patients bore a total financial burden of $412 direct costs and $3,146 indirect costs (total cost $3,558). The direct cost for diagnosis was $154 and for treatment was $258.	MDR-TB patients faced a substantial personal financial burden.
Furin et al. [16]	2001	Lima	Case study	60	Psychosocial adverse effects	The baseline depression rate was 50%, and 18.3% and 11.7% of patients newly developed depression or anxiety during the course of treatment, possibly as an adverse effect of medications.	Strategies to manage the neuropsychiatric adverse effects of MDR-TB therapy to increase adherence and treatment completion.
Vega et al. [7]	2004	Lima	Case study	75	Prevalence of depression, anxiety, andpsychosis	The baseline prevalence rates of depression and anxiety were 52% and 9%. During treatment, 13%, 12%, and 12% newly developed depression, anxiety, and psychosis respectively.	Continuation of TB drugs and administration of anti-depressant drugs together was thought to be an effective strategy for addressing MDR-TB medication-related psychiatric issues.

#### Effect of TB drugs on the psychological status of MDR-TB patients

It has been suggested that the side effects of MDR-TB drugs sometimes result in greater psychological problems than the disease. An evaluation study reported that the major adverse outcomes were neurologic side effects (depression, convulsions, consciousness, psychosis, suicide; 7%), especially due to the cycloserine [26]. One study found 50% baseline depression, 18% newly developed depression and 11% newly developed anxiety along with other physiological side effects of drugs among participants [16]. Two studies have reported that MDR-TB leads to inability to work due to treatment side effects and incapacitating depression at the time of diagnosis, resulting in loss of income and diminution of responsibilities [9,19]. Furthermore domestic abuse and suicidal temptation were reported as a consequence of MDR-TB [17,18].

### (2) Social issues among the MDR-TB patients

Eleven studies have investigated the social issues of MDR-TB patients. This included concerns around stigma and discrimination; impact of alcohol on MDR-TB management problems related to coinfection of MDR-TB with HIV and the economic issues related to MDR-TB.

#### Stigma and discrimination

Stigma has been reported as a major concern facing MDR-TB patients [7], Stigma in the context of MDR-TB negatively impacts the patient in accessing healthcare facilities in their neighbourhood [19]. The effects of stigma include experiences of social seclusion or rejection from family members, friends, neighbours, and/or health providers; internalized shame; financial instability; discrimination; and it repercussions [8]. One study among health care providers highlighted that the major barriers for the MDR-TB patients were social resulting from stigma rather than medical. Social acceptance and support were not present for MDR-TB, unlike other diseases [19]. It has also been reported that MDR-TB patients would voluntarily separate themselves from their family for fear of spreading infection to other members [9]. The impact of stigma reported has led to divorce, cancellation of impending marriages, breakdown of family relationships and also isolation within the family [22].

Alcohol has also proved detrimental to treatment completion among MDR-TB patients. A retrospective study on risk factors for default from MDR-TB treatment found that alcohol use was independently associated with treatment default (Hazard ratio 2.11; CI: 1.11–4.02; p = 0.02) [24].

The management of MDR-TB patients with HIV remains highly challenging due to the psychosocial issues resulting from co-infection. It has been found that HIV augments the already severe psychosocial burden faced by MDR-TB patients [19,20].

#### Economic issues

MDR-TB has a huge adverse economic impact on patients due to the long duration and complexity of treatment. The socioeconomic barriers include inaccessibility of treatment, distance, transport costs and costs incurred during hospitalization [25]. One study found that 23% of MDR-TB patients had defaulted on treatment due to financial constraints [23]. Another study reported that 5/10 patients had not resumed work even after one year of treatment, and at times caregivers also had to stop going to work for months at a time. There was also a reduction in salary due to work absenteeism and some income had to be spent on costs associated with treatment [21].

### (3) Psychosocial intervention studies

The interventions reported in the included studies were diverse; some were focused on the individual level, while others were group or family-focused interventions. The types of intervention included group therapy, personalized psychosocial support, recreational activities, symbolic celebrations, family workshops, therapeutic care and post treatment support. Of the 15 studies, 7 included interventions (Table 3). Of these, five [8,17,18,20,25] were qualitative studies that described the psychosocial benefits of specific programmatic models of MDR-TB care; however, they did not test psychosocial interventions using an experimental design. The other two studies [22,23] focused exclusively on psychosocial interventions. Notably, there were no high-quality randomized trials in the included studies. One study did compare two interventions either receiving counselling or financial incentives which was compared to a control group; however, it was a small pilot intervention. The other study had a quasi-experimental design.

**Table 3:** Intervention-based studies addressing psychosocial issues in MDR-TB patients.

Reference	Year	Place	Method	Popn.	Objective	Findings	Implications
Shin et al. [18]	2004	Lima	Qualitative	1000	Community based psychosocial model	Integrated approach with psychosocial, economical and medical support were very effective in controlling the epidemic set a blueprint for “complex health intervention in resource poor settings”	Community based psychosocial interventions for MDR-TB was extremely effective.
Chalco et al. [17]	2006	Lima	Qualitative	7 Nurses	Emotional support provided by nurses	Patients faced guilt, social stigma, adherence, side effects, socio-economic difficulties, special situations HIV surgery, domestic violence, treatment failure, family support issues for money, fear of return back to normal life post treatment.	Meetings with family, gatherings, therapeutic care and monitoring were effective
Acha et al. [8]	2007	Lima	Qualitative	285	Psychosocial support group intervention	Prevalence of stigma, depression, anxiety, suicidal tendencies, treatment side effects, loss productivity, hopelessness MDR treatment.	Group therapy, psychosocial support, recreations, symbolic celebrations and family workshops may be effective interventions; larger randomized trials are needed.
Horter et al. [25]	2014	Uganda	Qualitative(Intervention)	7	Acceptability & accessibility of home-based treatment	Home-based care was safe, conducive, psychosocially supportive & provide earning potential	Home based treatment is acceptable by all stake holders
Das et al. [20]	2014	India	Case study	45	Psychiatric conditions of HIV/MDR-TB patients	Baseline: Depression16%; (4/7 moderate to severe) After 3 months of therapy 9% no depression.	Individualized psychological & clinical support interventions are strongly recommended.
Kaliakbarova et al. [23]	2013	Kazakhstan	Cohort(Intervention)	426	Assess the effects of psychosocial support (PSS)	Pre PSS 23% had interrupted treatment due to financial reasons which reduced to 0.5% post due to alcohol addiction). 94% satisfied with the psychosocial support, 69% mentioned it as important.	PSS programme is successful in reducing default rate
Baral et al. [22]	2014	Nepal	Mixed(Intervention)	156	Impact of counseling; counseling with financial support	Extremely vulnerable to stigma and faced financial hardship. MDR-TB cure rate: Counselling 85%; counselling with finance 76%; no support 67% (differences not statistically significant)	Provision of counselling & financial support may increase cure rates given a trend towards statistical significance; however, larger studies are needed.

#### Descriptions of the programmatic intervention models of MDR-TB care

Home-based care by family members [25], emotional support from healthcare providers (including medical staff and nurses) [17], community based care such as involvement of community DOTS providers, peer group support [18], and financial support to patients [22] were interventions reported in these studies. The patients who had been provided with strong psychosocial and behavioural support along with the treatment showed a low default rate [19] and increased self-esteem [22], A rural study in Uganda [25] reported that home-based treatment for MDR-TB has benefits when compared to hospital-based treatment due to the increased psychosocial support available at home from family members, and avoidance of the financial costs associated with hospital admissions, Three of the five Peruvian studies [8,17,18] highlighted that engagement with families of MDR-TB patients was an essential part of the success of their integrated community-based interventions. These studies also provided economic support to MDR-TB patients as part of their interventions in the form of free treatment, as well as discounts on hospital costs and transportation costs. A Mexican study noted that free TB Treatment, transportation, and food assistance were helpful in improving treatment adherence in MDR-TB patients [9]. The need to address economic barriers was also reported in another study which suggested that support for housing and steady employment reduced the risk of default for MDR-TB treatment [24].

One intervention study for depression reported positive outcomes with the combination of antidepressant drugs with MDR drugs. There was an improvement from the base line depression rates to the follow up period for two thirds of the MDR patients who were part of this intervention [7]

In another study, 16% of MDR-TB patients co-infected with HIV were found to have depression at baseline. Individualized psychosocial and clinical support reduced depression at 3 months with depression being reported in 9% of the patients [20]. Other intervention studies included utilization of nurses, [8,9,17] other health workers [17], and cured MDR-TB patients [23] as key personnel in imparting psychosocial interventions such as counselling and support services in future intervention programmes. They also recommend incorporating psychosocial indicators into the standard MDR-TB patient monitoring protocols in national TB programmes [22].

Kaliakbarova et al. conducted a quasi-experimental study of a psychosocial support intervention in Kazakhstan. Provision of psychosocial support improved adherence to MDR-TB medications to 97%, as compared to only 48% treatment adherence prior to introduction of the intervention. Patient-oriented individual and group support programmes also showed success in reducing treatment default rates among MDR-TB patients [23].

Baral et al. conducted a pilot intervention in Nepal of counselling alone compared to counselling with provision of financial support. This study had a relatively high follow up rate of 80% and this intervention was linked to the treatment outcome of MDR-TB patients. The study found improvement in treatment outcomes in the intervention groups. MDR-TB cure rate in the group who received counseling alone was 85%; the cure rate in the group who received counseling with financial support was 76%; and cure rate in the group who received no support was only 67% [22].

### Quality of the evidence

Qualitative findings might be unique to the relatively small number of participants in these research studies but cannot be interpreted for wider populations. One third of the included studies in this review were from the same setting in Peru and the psychosocial aspects of care were included as components of a larger intervention programme [7,8,16,17,18]. The individual findings obtained from these studies could overlap and might be distinctive to that study setting and time period. The Peruvian studies were conducted during a period of MDR-TB outbreak to mitigate the MDR-TB epidemic. Such a “mitigation” model intervention and related research findings may not be suitable for other countries where MDR—TB has developed over decades (such as India). It was seen that the intervention studies had relatively high follow-up of patients; however, the sample sizes were small and the group allocation was not randomised. There is an urgent need for high-quality randomised controlled trials of psychosocial interventions for MDR-TB patients that focus on improving a wide variety outcomes, including treatment outcomes (e.g., cure), mental health, and quality of life.

## Discussion

This review reflects a relative dearth of MDR-TB research publications from low- and middle-income countries that have addressed the psychosocial issues challenging MDR patients. This is extremely important as this understanding and interventions that address these issues are important for the successful management of MDR patients as well as to pave the way for its control.

This review has highlighted the various psychosocial issues challenging MDR-TB patients caused because of the illness itself, as a side effect of the drugs as well as the stigma attached to this disease. Some studies also suggest that the psychological state of MDR-TB patients is shaped substantially by their earlier experiences of treatment failure on standard TB regimens, prior to their diagnosis of MDR-TB. These experiences of previous treatment failures lead to a feeling of doubt and emotional insecurity regarding the possibility of ever being cured of TB. While some of the psychosocial and economic issues can be addressed with the right interventions which do require further research. The psychological disturbances due to the drugs are a matter of concern. Those reported were neurologic side effects (depression, convulsions, consciousness, psychosis, suicide; especially due to the cycloserine [26]. Adverse side effects also include gastritis, dermatological effects, peripheral neuropathy and hearing loss [16]. These toxicities adversely affect health in many ways, particularly patient’s social, psychological health and quality-of-life. Ideally, the best therapeutic option would be to remove these drugs [28] that cause these psychological disturbances but in the case of MDR-TB, therapeutic options are limited and it is sometimes not possible to remove or substitute medications, despite their neuropsychiatric toxicity. It is also worrisome that the economic burden caused due to the illness, long duration of the treatment, hospitalization and the side effects of the drugs are often not addressed. It is important for more feasible, acceptable and affordable economic need based interventions to be tested in this regard to ensure adherence for MDR-TB treatment as well as for a better quality of life.

One of the significant barriers challenging MDR patients is stigma in its various forms from family, community and more importantly from health providers [29,30]. This is worrying and calls for psychosocial interventions to address these issues not only for the patients who suffers this stigma but it also emphasizes a need for sensitization among health care providers who play a major role in providing quality care. Furthermore sensitization efforts among the care givers and the community are crucial to facilitate the kind of support that patients require, especially with an illness that requires prolonged treatment. This has been reflected in a home-based intervention clinical management for MDR-TB was found to be effective and less expensive in African settings [31,32].

Stigma in the context of MDR-TB negatively impacts the patient in accessing healthcare facilities in their neighbourhood [19] and can result in TB transmission. These findings call for the patient centred approach and unless the patient concerns are addressed the challenges facing MDR-TB patients will continue and control of MDR-TB will be difficult. These concerns highlight the need for professional trained counsellors to equip MDR-TB patients to cope with the illness with need based psychosocial interventions at all levels from diagnosis till completion of treatment. They will also need to closely work with health providers, families and the community in TB care sensitization programmes as their support is crucial in dealing with a disease such as MDR-TB.

The study findings have also highlighted the economic burden due to MDR-TB with issues related to inaccessibility of treatment, distance, transport costs and costs incurred during hospitalization [33]. Inability to work due to the side effects of the drugs and therefore loss of income has been a major impediment leading to treatment default. WHO calculated that the average drug sensitive TB patient loses three to four months of work-time and up to 30% of their household annual earnings [34,35]. If this is the case for drug sensitive TB, one can imagine the huge burden for MDR-TB patients. Furthermore these economic and financial challenges have a broader psychological impact on MDR patients and their families throughout the disease course. This calls for the need to consider incentivizing MDR patients during treatment which could help tide over the financial crisis for patients caused by the illness and related psychological distress. Financial support can also enable people in terms of affordability, access to care and promote adherence. In addition this may help to improve nutritional intakes and thereby reduce vulnerability.

Studies have also reported alcohol abuse as one of the risk factors for MDR-TB [36] the link between alcohol abuse and MDR-TB management may not be a direct causal relationship [37,38]. Instead, MDR-TB may be the result of interruptions in treatment resulting from alcohol use [39]. Treatment default is sometimes attributed to psychosocial problems resulting from alcohol use [9,40]. This can also be due to prior, pre-TB psychological conditions that pre dispose TB disease especially MDR-TB. There is a need for effective alcohol intervention strategies address alcohol abuse among the MDR-TB patients to bring about better treatment compliance [16].

The review also suggests that the psychosocial problems faced by HIV/MDR-TB co-infected patients are particularly challenging. Studies show that the mortality due to MDR-TB among the HIV infected patients is unusually high [41,42]. While the use of antiretroviral therapy (ART) with MDR-TB treatment has resulted in improved outcomes in co-infected patients [43,44,45], the management of MDR-TB patients with HIV remains highly challenging due to the psychosocial issues around this co-infection. The risk factors for HIV and MDR-TB are largely overlapping [46]. Issues regarding adherence to treatment for both these diseases remain a challenge as the duration of treatment is long or indefinite for both diseases. One study have shown that there is preferential adherence to the ART drugs compared to TB drugs due to the pill burden and social morbidity associated with it. [47] High quality TB control programmes are successfully managing patients with HIV and drug-susceptible TB. The same successful approach has to be scaled up for HIV/MDR-TB co-infected patients also. There is no well-defined patient centred approach in TB paradigm for MDR-TB. Directly Observed Treatment is the only strategy is available to provide some support to the drug sensitive TB patients and there is no provision addressing psychosocial issues under the programme condition. Perhaps we need to learn from HIV control programmes where counsellors are an integral part of the treatment initiation and management that include pre-test and post-test counselling as well as subsequent regular counselling for patients and their families during the course of the treatment.

In summary psychological issues were more severe at diagnosis and during the early stages of the disease. The social issues relate to the family and community of the patient may persist throughout the disease course, sometimes even after the disease ceases. Involvement of family, community in the disease management was found to be one of the ways to overcome the social issues like stigma and discrimination while also improving treatment outcomes. Economic and financial challenges also affect patients and their families throughout the disease course, especially because treatment costs accrue during the long duration of treatment. While several studies suggest that psychosocial interventions may have a positive impact on the lives and treatment outcomes of MDR-TB patients, the quality of these studies is low. Also, some of the studies did not have standardised protocols for their interventions. Our rating of the quality of these studies highlights a need for more randomized trials. In addition, future studies need to evaluate whether psychosocial interventions have an impact on long-term treatment outcomes. Some of the studies recommend that national TB control programmes should collect data on key psychosocial indicators are part of their MDR-TB monitoring framework. The importance of counsellor and the influence of counselling in the intervention cannot be overstated [16,48].

### Limitation of this review

Despite a thorough literature search, the studies included are relatively small in number, which is a major limitation of the review. Another limitation is that we did not include unpublished studies and studies published in non-indexed journals. The heterogeneity of the included studies in terms of study design, study variables and outcome measures limited the scope for synthesising the data and further interpretation, including meta-analysis. The qualitative methods and tools reported in the studies were diverse and locally appropriate. Quantitative tools used in a few studies to measure the psychosocial morbidity were not validated or pretested which raises questions about methodological quality. We cannot draw firmer conclusions from this review given limitations mentioned.

## Conclusion

Our review highlights the serious psychosocial challenges experienced by MDR-TB patients both due to disease and its treatment complexities. The limited number of published studies on this vital topic underscores a need for greater investment in research focusing on psychosocial status of MDR-TB patients. We observed that the limited psychosocial and economic interventions reported in these studies suggest improvements in both treatment adherence and cure rates among MDR-TB patients. However there is an urgent need to build upon these preliminary findings to generate more evidence with large scale multicentre studies randomised control studies to provide information on proven psychosocial interventions that can be replicated in the TB control programmes.

## Supporting Information

S1 Table PRISMA 2009 Checklist. (DOC)

S2 Table List of 35 excluded studies and reasons for exclusion. (DOC)
