# Supplementary material for: Psycho-Socio-Economic Issues Challenging Multidrug Resistant Tuberculosis Patients: A Systematic Review
Source: PLoS One. 2016 Jan 25;11(1):e0147397. doi: 10.1371/journal.pone.0147397 (PMC4726571; doi:10.1371/journal.pone.0147397)
Supplement: S2 Table — (DOC) [file pone.0147397.s002.doc]

**S2 Table. List of 35 excluded studies and reasons for exclusion**

| **No** | **Author** | **Title** | **Reason for exclusion** |
| --- | --- | --- | --- |
| 1 | Sonya Shin | Adverse reactions among patients being treated for MDR-TB in Tomsk, Russia | Non psychosocial issue |
| 2 | Carole D Mitnick | Aggressive regimens for multidrug-resistant tuberculosis decrease all-cause mortality | Non psychosocial issue |
| 3 | Caroline Frank | Assessing the impact of multidrug-resistant tuberculosis in children: an exploratory qualitative study | Participants were Children |
| 4 | Cesar Urate Gil | Association of major depressive episode with negative outcomes of tuberculosis treatment | Focus was on treatment outcomes |
| 5 | Padayatchi N | Case series of the long-term psychosocial impact of drug-resistant tuberculosis in HIV-negative medical doctors | Participants were health care providers |
| 6 | Davies PD | Drug-resistant tuberculosis: concluding remarks | Non psychosocial issue |
| 7 | Libo Liang | Factors contributing to the high prevalence of multidrug-resistant tuberculosis: a study from China | Non psychosocial issue |
| 8 | Floyd K | Financial resources required for tuberculosis control to achieve global targets set for 2015 | Non psychosocial issue |
| 9 | Tudor C | Health care workers' fears associated with working in multidrug- and or extensively-resistant tuberculosis wards in South Africa | Participants were health care providers |
| 10 | Shona Horter | I can also serve as an inspiration": a qualitative study of the TB & Me blogging experience and its role in MDR-TB treatment | Non psychosocial issue |
| 11 | [Gustavo E Velásquez](http://cid.oxfordjournals.org/search?author1=Gustavo+E.+Velásquez&sortspec=date&submit=Submit) | Improving outcomes for multidrug-resistant tuberculosis: aggressive regimens prevent treatment failure and death | Non psychosocial issue |
| 12 | ZF Udwadia | India’s multidrug-resistant tuberculosis crisis | Non psychosocial issue |
| 13 | Fraser HS | Informatics tools to monitor progress and outcomes of patients with drug resistant tuberculosis in Peru | Focus was on treatment outcomes |
| 14 | HS Fraser | Information systems for patient follow-up and chronic management of HIV and tuberculosis: a life-saving technology in resource-poor areas | Non psychosocial issue |
| 15 | Brust JCM | Integrated, home-based treatment for MDR-TB and HIV in rural South Africa: an alternate model of care | Unable to retrieve data |
| 16 | Farmer P | Management of MDR-TB in resource-poor countries | Unable to retrieve data |
| 17 | Thomas A | Management of multi drug resistance tuberculosis in the field: Tuberculosis Research Centre experience | Non psychosocial issue |
| 18 | Christoph Lange | Management of patients with multidrug-resistant/extensively drug-resistant tuberculosis in Europe: a TBNET consensus statement | Participants were health care providers |
| 19 | Marica C | Multidrug resistant tuberculosis in Romania in the last years (2004-2007)--an extremely important social phenomenon | Focus was on treatment outcomes |
| 20 | [**Escudero E**](http://www.ncbi.nlm.nih.gov/pubmed/?term=Escudero E%5BAuthor%5D&cauthor=true&cauthor_uid=16602405) | Multidrug-resistant tuberculosis without HIV infection: success with individualized therapy | Unable to retrieve data |
| 21 | [**Chonde TM**](http://www.ncbi.nlm.nih.gov/pubmed/?term=Chonde TM%5BAuthor%5D&cauthor=true&cauthor_uid=20626940) | National anti-tuberculosis drug resistance study in Tanzania | Non psychosocial issue |
| 22 | Hind Satti | Outcomes of comprehensive care for children empirically treated for multidrug-resistant tuberculosis in a setting of high HIV prevalence | Participants were Children |
| 23 | P Isakidis | Poor outcomes in a cohort of HIV-infected adolescents undergoing treatment for multidrug-resistant tuberculosis in Mumbai, India | Non psychosocial issue |
| 24 | Amrita Daftary | Preferential adherence to antiretroviral therapy over tuberculosis treatment: a qualitative study of drug-resistant TB/HIV co-infected patients in South Africa | Non psychosocial issue |
| 25 | Furin J | Programmatic management of multidrug-resistant tuberculosis: models from three countries | Non psychosocial issue |
| 26 | [**Joia S Mukherjee**](http://www.sciencedirect.com/science/article/pii/S0140673604154962) | Programmes and principles in treatment of multidrug-resistant tuberculosis | Non psychosocial issue |
| 27 | [**Becerra MC**](http://www.ncbi.nlm.nih.gov/pubmed/?term=Becerra MC%5BAuthor%5D&cauthor=true&cauthor_uid=10815730) | Redefining MDR-TB transmission 'hot spots' | Non psychosocial issue |
| 28 | [**Mark Daku**](http://www.sciencedirect.com/science/article/pii/S0277953612002262) | Representations of MDR and XDR-TB in South African newspapers | Non psychosocial issue |
| 29 | Atun R | Resistance to implementing policy change: the case of Ukraine | Non psychosocial issue |
| 30 | Keshavjee S | Treating multidrug-resistant tuberculosis in Tomsk, Russia: developing programs that address the linkage between poverty and disease | Non psychosocial issue |
| 31 | Sonya Shin | Treatment of multidrug-resistant tuberculosis during pregnancy: a report of 7 cases | Non psychosocial issue |
| 32 | [**Yanina Balabanova**](http://bmjopen.bmj.com/search?author1=Yanina+Balabanova&sortspec=date&submit=Submit) | Survival of drug resistant tuberculosis patients in Lithuania: retrospective national cohort study | Non psychosocial issue |
| 33 | SS Shin | Treatment outcomes in an integrated civilian and prison MDR-TB treatment program in Russia | Non psychosocial issue |
| 34 | Becerra, Mercedes C | Tuberculosis in children exposed at home to multidrug-resistant tuberculosis | Participants were Children |
| 35 | Hopkins L | When control measures fail | Participants were health care providers |
